# Supplementary material for: Efficiency Improvement Strategies for Public Health Systems: Developing and Evaluating a Taxonomy in the Australian Healthcare System
Source: Healthcare (Basel). 2023 Jul 31;11(15):2177. doi: 10.3390/healthcare11152177 (PMC10419221; doi:10.3390/healthcare11152177)
Supplement: Supplementary file 1 [file healthcare-11-02177-s001.zip › healthcare-2491667-supplementary.pdf]

health.nsw.gov.au

NSW Health Program Management Office

Summary of expenses improvement strategies

2016-17 to 2018-19 Financial Years

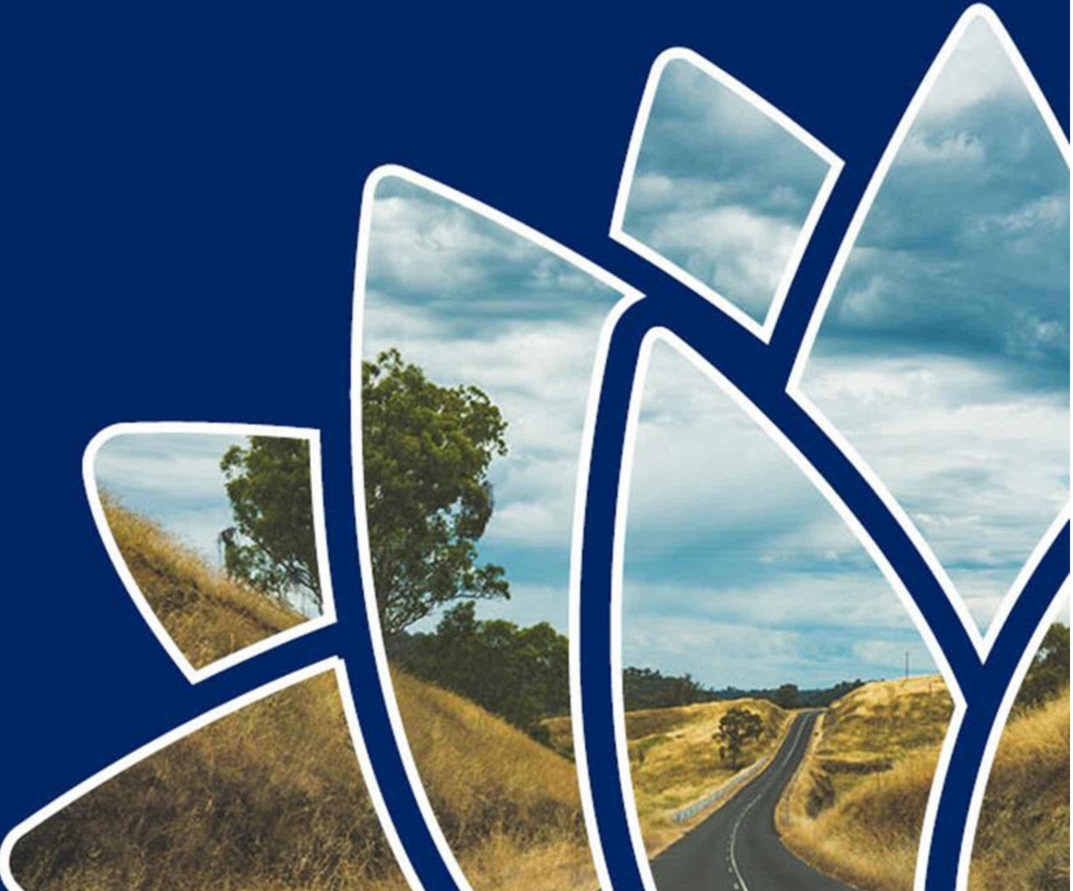

NSW GOVERNMENT

Summary of NSW Health Expenses improvement strategies: Support Resource Document

NSW Health organisations undertake efficiency improvement plans which are aimed at reducing expenditure each Financial Year. The NSW Health Program Management Office has undertaken a complete review of expenses reduction strategies supported through Roadmapping during the 2016/17, 2017/18 and 2018/19 Financial Years. Each unique approach to reducing expenditure which was identified has been summarised in this document. Similar strategies which were identified multiple times during the review process are presented in this review as a single entry to avoid duplication. Out of the 1,127 expenses Roadmaps supported during this timeframe a total of 263 unique expenses reduction approaches were identified.

This document provides a high-level summary of the means by which each of these approaches generated an expenses reduction, along with the way in which expenses improvements were calculated. Strategies have been grouped according to their category of savings for ease of reference. This resource is intended to be used as an ideas generation guide for NSW Health Organisations seeking to identify opportunities to reduce expenditure. Although this resource provides a complete historical perspective of contemporary savings initiatives across NSW Health, it remains important for NSW Health organisations to also consider innovative solutions and future opportunities to generate savings in new ways. It is also important to note that NSW Health Organisations must consider which savings approaches are most appropriate to their specific local needs.

Further information regarding any of the strategies summarised in this resource may be obtained by contacting the PMO team at HSSG-PMO@health.nsw.gov.au

NSW GOVERNMENT | Health

Clinical contracts & supplies

Strategy	Benefit measurement
Purchase equipment from trust fund rather than general fund	Total trust funds released
Reduce pharmacy stock write-off through better stock management	Baseline stock write-off cost - current stock write-off cost
Obtain external funding for internally-funded care	Total value of funding covered by external source
Terminate external service contract and use existing internal capabilities	Total contract cost over period cancelled
Standardise and consolidate products in use	Baseline stock cost – current stock cost
Change to a more cost-effective supplier	Baseline contract cost - renegotiated contract cost
Negotiate discount for bulk purchase	Baseline contract cost - renegotiated contract cost
Reduce externally-provided document scanning into eMR services	Baseline scanning expenditure - current scanning expenditure
Review and prioritise routine maintenance and delay non-essential works	Baseline expenditure - current expenditure
Install solar panels as an alternative to mains power	Mains power cost - solar cost + cost of solar panel installation
Reduce excess stock, enhance stock rotation, centralise catalogue	Baseline stock expenditure - current stock expenditure
Reduce pharmacy stock volume	Baseline pharmaceutical expenditure - current pharmaceutical expenditure
Consolidate procurement processes	Baseline expenditure - current expenditure
Reduce linen expenses through eliminating inefficient practices	Baseline expenditure - current expenditure
Provide services on-site instead of outsourcing	Total fees saved for non-outsourced services
Contract renegotiation, contract consolidation and contract management enhancement	Baseline contract expenditure - current contract expenditure
Reduce waste of blood products through CEC Blood Watch program	Units of avoided waste x cost per unit
Reduce procurement of non-catalogue items, enhance short date product management	Baseline consumables expenditure - current consumables expenditure
Identify more cost-effective supply options for non-contract pharmaceuticals	Baseline non-contract supply expenditure - current non-contract supply expenditure
Enhance prescribing and ordering practices to reduce stocking-related inefficiencies	Total savings in stock management, procurement, storage and waste
Investigate less-costly alternatives for high cost and brand name drugs	Cost difference x number of units substituted
Improve linen usage through distribution enhancements and review of high-usage areas	Baseline linen expenditure - current linen expenditure
Enhance procurement governance, staff skills and support resources	Baseline consumables expenditure - current consumables expenditure
Target reduction in use of consumables for high-use areas	Baseline consumables expenditure - current consumables expenditure

Clinical contracts & supplies (continued)

Strategy	Benefit measurement
Standardise nutritional supplements through HealthShare as opposed to staff ordering	Baseline supplement expenditure - HealthShare cost
Purchase equipment as an alternative to rental	Difference between rental and purchase costs x units purchased
Identify and reduce high-cost consumable use	Baseline consumables expenditure - current consumables expenditure
Identify and reduce medication procurement outside of existing contracts	Total reduction in non-contract medication expenditure
Reduce price variation between facilities and standardise products	Baseline consumables expenditure - current consumables expenditure
Reduce wasted and expired stock by better stock management and staff engagement	Total reduction in wasted and disposed-of stock
Manufacture compounds rather than purchase	Purchase price - total manufacturing price x units
Reduce options available for ordering, enhance stock control and ordering practices	Baseline supplies expenditure - current supplies expenditure
Enhance management of linen to reduce non-essential usage	Previous linen expenditure - current linen expenditure
Use disposable surgical packs rather than reusable bowls and individually-wrapped items	Disposables cost - sterilisation cost x volume of products
Review prosthetics use and ensure cost-effective prosthetic product selections	Baseline prosthetics expenditure - current prosthetics expenditure
Change to a less-expensive product	Difference in product cost x units used
Tighten approval process for supplies ordering	Baseline consumables expenditure - current consumables expenditure
Switch from reusable to disposable surgical drapes	Disposables cost - sterilisation cost
Renegotiate equipment lease prices and remove end-of-term balloon payments	Total reduction in lease expenditure
Improve management of all waste types through education, technology and sustainable products	Total reduction in waste expenditure
Review medicines usage and change non-PBS items to PBS items	Total reduction in non-PBS expenditure
Negotiate multi-year contracts to prevent price increases in future years	Total increases avoided
Reduce paper medication chart costs through electronic medication management	Value of reduction in paper chart purchasing
Reduce advisory board expenditure by ceasing subscriptions	Total value of subscriptions ceased
Implement just-in-time ordering to reduce inventory and centralise stock storage	Total reduction in supply chain costs
Reduce supply use by reviewing usage practices	Total reduction in clinical supply expenditure
Streamline procurement practices to reduce transaction costs	Transaction cost x number of transactions avoided

NSW GOVERNMENT | Health

Clinical contracts & supplies (continued)

Strategy	Benefit measurement
Commence sustainable long-term replacement program for high-value medical equipment	Current purchase and maintenance costs - sustainable program costs
Audit theatre prosthetic and consumable waste and reduce waste where possible	Total value of waste reduced
Reduce home Total Parenteral Nutrition expenses by reviewing ordering practices	Total reduction in TPN expenditure
Renegotiate high-cost drug prices	Baseline contract cost - renegotiated contract cost x volume of drugs ordered
Lease new equipment to avoid repairs and maintenance costs on existing equipment	Repairs and maintenance costs - lease costs
Streamline ordering process through barcoding and product standardisation	Total reduction in supply chain costs
Change from brand name to generic medications	Brand cost - generic cost x number of units
Review weekend pathology use and address overuse where identified	Total reduction in pathology costs
Reduce external medical imaging reporting by using internal medical staff where appropriate	Cost per external report x number of reports reduced
Review rented equipment in comparison to need and reduce where possible	Total reduced rental expenses

Clinical service delivery & Patient Outcomes

Strategy	Benefit measurement
Reduce staff training in excess of requirements	Baseline training expenditure - current training expenditure
Seasonal shutdown	Routine period cost - shutdown period cost
Cease on-call service	Total cost of service ceased over period
Reduce medical imaging where clinically appropriate	Baseline imaging requests - current imaging requests x cost per image
Reduce diagnostic procedures where clinically appropriate	Baseline pathology expenditure - current pathology expenditure
Reduce elective procedures (hip and knee replacement) where clinically appropriate	Number of reduced procedures x procedure cost
Reduce avoidable patient transport	Baseline transport cost - current transport cost
Enhance patient transport scheduling to reduce after-hours transportation rates	Cost difference between standard and after hours transport x number of transports
Extend service hours to reduce after-hours call-backs	Reduction in call-backs x cost per call back - additional staff investment
Reduce surge bed use and allied health backfill during seasonal slowdown	Baseline expenditure - current expenditure
Reduce high-cost procedures through procedures occurring in alternate settings	Cost per procedure x number of procedures reduced
Cease providing meals and supplies to patient relatives	Meals reduced x cost per meal
Purchase additional transport vehicle to reduce transport and accommodation costs	Baseline costs - current costs including purchase and operating costs for new vehicle
Use pre-filled electronic registration forms and electronic GP communication	Cost per printed form and postage x number of electronic forms sent
Reduce patient post-discharge taxi usage where appropriate	Total reduction in taxi expenditure
Reduce Arterial Blood Gas tests where clinically appropriate	Number of tests reduced x cost per test
Renew clinical maintenance contracts and change to more cost-effective options	Baseline contract cost - renegotiated contract cost
Reduce pathology ordering where clinically appropriate	Baseline pathology expenditure - current pathology expenditure
Enhance ambulance use planning and ordering practices to reduce transport premiums	Baseline transport cost - current transport cost
Implement after-hours nurse-initiated contrast instead of using on-call radiology technician	Total on-call costs reduced
Enhance theatre utilisation to reduce elective surgery outsourcing costs	Reduction in outsourced procedures x costs per procedure types
Reduce diagnostic outsourcing	Baseline external diagnostics expenditure - current external diagnostics expenditure

Clinical service delivery & Patient Outcomes (continued)

Strategy	Benefit measurement
Review contractor/agency usage and realign with service needs to reduce excess use	Baseline agency/contractor costs - current agency/contractor costs
Standardise prosthetic product use in alignment with best clinical practice	Total reduction in prosthetics expenditure compared to baseline
Reduce procedure numbers to align with number of funded procedures	Number of unfunded procedures reduced x cost per procedure
Increase pre-booking of patient transport	Same-day booking cost - early booking cost x number of extra early bookings
Reduce label and medical record costs through electronic documentation	Total reduction in paper and label costs
Switch to digital dentistry to reduce material and manufacturing costs	Difference in manufacturing costs x units manufactured
Delay after-hours surgeries where clinically appropriate to reduce call-in penalties	Baseline call-in penalties - current call-in penalties
Use telehealth as an alternative to patient transport	Number of transports avoided x cost per transport + telehealth costs
Reduce pathology ordering in high-variance areas and provide ordering guidance to staff	Baseline pathology expenditure - current pathology expenditure
Develop clinical order sets to reduce unnecessary pathology testing	Baseline pathology expenditure - current pathology expenditure
Benchmark pathology use against peer facilities to identify areas for potential improvement	Total reduction in pathology expenditure compared to previous benchmark
Reduce "did not attend" instances in clinics through enhanced appointment reminders	Total reduced non-attendances x cost per non-attendance

Finance & operations

Strategy	Benefit measurement
Reduce discretionary travel and accommodation	Baseline expenditure - current expenditure
Close auxiliary services e.g. cafeteria	Total service cost
Consolidate to a single fleet maintenance service provider	Previous maintenance expenditure - current expenditure
Discontinue redundant natural gas and electricity connections	Annual cost of discontinued services
Change from outsourced to HealthShare-provided soft facility management services	Cost difference between outsourced and HealthShare-provided services
Outsource non-profitable services	Previous operating costs - contract cost
Audit for GST overpayments and recoup	Total funds recouped
Replace facilities and equipment to eliminate maintenance costs	Maintenance costs - purchase costs
Identify and cease any payments for goods not used, not received or used by non-health entity	Total value of payments reduced
Identify and reduce low-value equipment	Total annual cost of equipment discontinued
Change from paper-based literature and textbooks to electronic sources	Difference in subscription and maintenance costs
Manage accommodation bookings centrally rather than allowing staff to book accommodation	Total reduction in accommodation expenditure
Use electronic communications rather than post	Total reduction in print and postage expenditure
Reduce discretionary spending on travel, media and education	Baseline discretionary spending - current discretionary spending
Review property lease and rental arrangements and do not renew if unnecessary	Total value of any discontinued leases
Reduce VMO consultations and on-call	Baseline VMO expenditure - current VMO expenditure
Move service from rented premises to on-site premises	Annual rent saved
Better utilise existing capacity rather than opening surge beds	Total reduction in surge bed expenditure
Cease providing non-profitable support/ancillary service	Total operating loss in previous period and cost of service saved
Enhance financial accountability by identifying high-variance cost centres and targeting support	Total improvement in budget performance in targeted cost centres
Amalgamate IT systems	Cost of existing systems - cost of single new system + transition costs
Audit for overpayments, recover repayments and address recurrent issues	Value of overpayment reductions and total value of recovered overpayments
Enhance energy efficiency practices e.g. turning off equipment, staff training	Baseline utilities expenditure - current utilities expenditure
Enhance workers compensation practices	Total workers compensation related cost reduction

Leave management

Strategy	Benefit measurement
Reduce excess ADO payments for JMO's	Baseline ADO payments - current ADO payments
Reduce excess annual leave liability	Total value of excess leave hours reduced
Reduce sick leave	Baseline expenditure - current expenditure
Reduce excess annual leave by identifying issue areas and targeting support	Total reduced leave hours x average cost per hour
Reduce sick leave by identifying issue areas and targeting support	Total reduced leave hours x average cost per hour
Identify high sick leave users and commence performance management	Total reduction in sick leave hours x cost per hour
Reduce sick leave through staff and visitor education	Baseline sick leave cost - current sick leave cost
Do not backfill short-term non-frontline leave	Total value of hours not replaced
Enhance leave planning to minimise coverage costs	Baseline leave cost - current leave cost

Non-clinical contracts & supplies

Strategy	Benefit measurement
Renegotiate White Pages listing annual fee	Baseline contract cost - renegotiated contract cost
Expenses no longer incurred (Water rates)	Total annual rates expense
Full review of all contracts that have expired and renegotiate rates	Baseline contract cost - renegotiated contract cost
Reduce excess fleet vehicles	Number of reduced vehicles x vehicle cost
Reduce external consultants	Baseline external consultant expenditure - current external consultant expenditure
Streamline routine maintenance processes	Baseline maintenance costs - current maintenance costs
Reduce unnecessary stationery expenses	Baseline expenditure - current expenditure
Reduce energy costs through turning off appliances after hours	Baseline utilities expenditure - current utilities expenditure
Use on-site accommodation for short-term overnight staff accommodation	Total avoided accommodation charges
Change fleet vehicle provider	Annual contract cost difference
Reduce number of fleet vehicles	Cost per vehicle x vehicles reduced
Bulk order stationary and printing based on annual demand	Difference in cost per unit x units ordered
Renegotiate telecommunications contract	Baseline contract cost - renegotiated contract cost
Renew fleet vehicle leases to more cost-effective options	Baseline lease cost - current lease cost
Install solar panels to reduce utilities cost	Reduced cost + initial investment and maintenance costs
Reduce consultants and executive coaches	Reduced expenditure compared to baseline
Reduce printing	Pages per year reduced x cost per page
Cease/reduce external consultants	Total reduction in expenditure on external consultants
Reduce consultants, legal and membership costs	Total value of professional services reduced
Review contracted cleaning arrangements and reduce any over-servicing	Total reduction in contract cleaning service costs
Establish total asset management contracts rather than multiple separate contracts	Baseline contract cost - renegotiated contract cost
Enhance maintenance approval processes to reduce non-essential expenditure	Baseline maintenance costs - current maintenance costs
Enhance approval processes for IT equipment purchasing	Total reduction in IT purchasing compared to baseline
Remove excess desktop printers	Number of printers reduced x annual cost per printer

Premium staffing

Strategy	Benefit measurement
Reduce JMO rostered overtime	Baseline rostered overtime spend - Updated rostered overtime spend
Reduce ED locum placements	Baseline locum expenditure - current locum expenditure
Recruit to vacant positions as an alternative to overtime	Overtime hourly cost - regular hourly cost x hours recruited to
Reduce JMO overtime through better scheduling	Baseline overtime expenditure - current overtime expenditure
Reduce VMO activity in excess of contracted activity	Excess activity reduced x cost of activity
Reduce overtime by reducing sick leave	Baseline expenditure - current expenditure
Reduce locums through recruitment	Baseline locum expenditure - current locum expenditure + recruited staff costs
Reduce overtime through rostering, staffing, recruitment and leave planning	Baseline overtime expenditure - current overtime expenditure
Reduce overtime in excess of nursing hours per patient day	Baseline overtime expenditure - current overtime expenditure
Change VMOs from fee-for-service to sessional contracts	Baseline VMO expenditure - current VMO expenditure
Replace contract staff with permanent staff	Reduction in contract staff expenditure + additional staff cost
Reduce overtime through better governance of overtime processes	Baseline overtime expenditure - current overtime expenditure
Reduce call-backs for unnecessary diagnostic procedures	Baseline expenditure - current expenditure in call-backs and in diagnostic costs
Improve rostering compliance with awards to reduce penalty rates	Baseline penalty rate expenditure - current penalty rate expenditure
Recruit to vacancies, enhance leave planning, optimise casual pool	Baseline penalty rate expenditure - current penalty rate expenditure
Reject VMO payments which do not comply with policy requirements	Total value of rejected payments
Reduce overtime by enhancing governance of overtime approval	Baseline overtime expenditure - current overtime expenditure
Upskill registrars to reduce reliance on overtime related to need for enhanced skillsets	Baseline medical overtime - current medical overtime
Forward roster planning to mitigate locum and VMO requirements	Baseline staff expenditure - current staff expenditure
Audit non-rostered medical overtime and address underlying causes	Baseline medical overtime - current medical overtime
Enhance rostering, annual leave, sick leave and casual staff management to reduce overtime	Baseline overtime expenditure - current overtime expenditure
Identify and target staff with patterns of concurrent overtime and sick leave use	Reduced leave hours x cost per hour

Premium staffing (Continued)

Strategy	Benefit measurement
Reduce number of nursing agencies used in favour of most cost-effective agency	Previous agency expenditure - current agency expenditure
Increase new graduate intake into vacant roles to reduce reliance on premium labour	Baseline premium staffing expenditure - current premium staffing expenditure
Centralise approval of overtime to maximise use of existing alternatives	Baseline overtime expenditure - current overtime expenditure
Redeploy rostered-on staff from lower-capacity units to areas of clinical need	Number of redeployments x cost of replacing shift with additional staffing
Redesign JMO roster to reduce JMO overtime	Baseline overtime expenditure - current overtime expenditure
Reduce late submission of VMO claims and increase governance of claims process	Baseline VMO expenditure - current VMO expenditure
Reduce non-clinical overtime by implementing non-clinical overtime approval process	Total reduction in non-clinical overtime
Roster as per Health roster guidelines to reduce overtime and payroll adjustments	Total reductions in overtime and payroll adjustment payments
Recruit to vacant positions to reduce agency staff use	Total reduction in agency staff costs
Renegotiate agency staff contract with provider to reduce overall costs	Total reduction in hourly rate x hours booked
Review rostering and staff management to reduce penalty payments	Baseline penalty rate expenditure - current penalty rate expenditure
Reduce contingent staffing in non-essential roles	Total reduction achieved in contingent labour costs
Convert VMOs to Privately-Referred Non-Inpatient Billing where appropriate	Total difference in billing types

Recruitment, vacancies & FTE

Strategy	Benefit measurement
Do not backfill vacant position	Establishment x time vacant
Reduce establishment for temporary staff	Baseline establishment hours - current establishment hours x cost per hour
Recruit to vacant positions as an alternative to temporary/agency staff	Contract staff cost - permanent staff cost
Reduce VMO sessional contracts	Baseline VMO expenditure - current VMO expenditure
Voluntary redundancies	FTE reduced + redundancy costs
Reduce locums through JMO recruitment	Baseline locum expenditure - current locum expenditure + JMO costs
Enhance retention strategies	Baseline recruitment and vacancy costs - current expenditure
Establish medical training positions to enhance recruitment and reduce overtime	Baseline recruitment and vacancy costs - current expenditure
Delay recruitment to vacant positions where service delivery not impacted	Time vacant x cost of position grade
Backfill leave with reduced staff grades where clinically appropriate	Difference in hourly rate x hours replaced
Maintain established positions unfilled without impacting service delivery	Total cost of unfilled positions
Reduce FTE through voluntary redundancies	Total cost of reduced positions
Reduce workers compensation premiums through better claims management	Total reduction in premiums achieved
Renegotiate private practice levels for staff specialists	Total reduction in staff specialist expenditure
Recruit to vacant medical positions to reduce locum expenditure	Annual difference in medical staff expenditure and locum staff x positions filled
Hire staff to manage outsourced labour internally	Previous contract cost - staff hire cost
Employ new graduate nurses instead of experienced higher grade nurses	Difference in hourly rate x hours per year x number of new graduates hired

Staffing Models

Strategy	Benefit measurement
Increase clinical split in management/clinical roles	FTE reduction achieved through increasing clinical component of management roles
Roster staff coverage to reduce call-out expenditure	Baseline callout rate - current callout rate x cost per callout
Reduce travel costs by lengthening shifts	Baseline travel expense - current travel expense
Reduce VMO requirements through nurse-delegated emergency care	Baseline VMO expenditure - current VMO expenditure
Reduce patient specials where avoidable	Baseline patient specialling expenditure - current expenditure
Use local GPs instead of locums in the emergency department	Baseline locum expenditure - current locum expenditure
Reduce FTE by balancing rostering with activity & demand	Baseline cost of FTE allocated - current cost of FTE allocated
Improve governance processes to provide staffing levels in line with activity	Baseline staff expenditure - current staff expenditure
Improve cross-hospital partnerships with patient flow, team leaders and Emergency	Baseline staff expenditure - current staff expenditure
Amend model to meet clinical need with reduced staff travel, accommodation and premiums	Baseline expenditure - current expenditure
Reduce after-hours rostered staff where clinically appropriate	Total reduced hours x cost per hour
Restructure service to reduce need for staff allowances	Total allowance costs reduced
Centralise clinical services to reduce need for staffed outlier beds	Total outlier staffing expenditure
Replace VMOs with Staff Specialists	Cost difference between provider types x providers replaced
Introduce skill mix-based staffing to balance Registered, Enrolled and Assistants in Nursing	Previous staffing expenditure - current staffing expenditure
Use assistants in nursing for patient special assignment rather than security guards	Difference in cost x hours allocated
Fill vacant positions with new graduate nurses rather than high-grade registered nurses	Difference in hourly rate x hours per year x number of new graduates hired
Use nurse-delegated emergency care instead of medical staff where appropriate	Difference in staff expenditure
Merge roles/positions	Difference in employee-related expenditure after merging roles
Discontinue vacant support roles and reallocate workload to existing positions	Total annual cost of discontinued roles
Redesign roster to meet clinical need with less-expensive medical staff	Baseline roster cost - current roster cost

NSW GOVERNMENT | Health

Staffing Models (Continued)

Strategy	Benefit measurement
Develop casual support staff pool to reduce overtime for support services	Reduction in support services costs
Add an additional after-hours shift to reduce overtime	Reduced overtime + cost of additional shift
Review non-clinical vacancies as they occur and revise roles where appropriate	Total reduction in FTE value
Transfer in-house services to shared services	Total reduction in service costs
Recruit lower-grade staff to vacant positions while maintaining service delivery	Difference between establishment and lower-grade annual costs
Convert allied health to allied health assistants	Difference in annual establishment cost x positions replaced
Review rosters and reduce overstaffing where identified	Total value of overstaffing avoided

NSW
GOVERNMENT
